# Prenatal suspicion and ultrasound diagnosis of umbilical artery thrombosis: A case report with targeted Doppler evaluation and timely delivery

**DOI:** 10.1097/MD.0000000000043776

**Published:** 2025-08-15

**Authors:** Gang Li, Xiufeng Yin, Jianfeng Guo, Yeping He

**Affiliations:** a Department of Ultrasound, The Affiliated Yixing Hospital of Jiangsu University, Wuxi, Jiangsu Province, China; b Department of Gynaecology and Obstetrics, The Affiliated Yixing Hospital of Jiangsu University, Wuxi, Jiangsu Province, China.

**Keywords:** hemodynamic changes, high resolution flow, ultrasound, umbilical artery thrombosis, umbilical cord

## Abstract

**Rationale:**

Umbilical artery thrombosis (UAT) is a rare complication that is strongly associated with adverse neonatal outcomes, and the diagnosis and selection of an appropriate delivery time for good neonatal outcomes is challenging. Here, we present a case of prenatal suspicion and ultrasound diagnosis of UAT: a case report with targeted Doppler evaluation and timely delivery.

**Patient concerns:**

A 35-year-old (gravida 6, para 1) with reduced fetal movement at 32 weeks gestation, Doppler ultrasound showed that for gestational age, the pulsatility index (PI), resistance index, and peak systolic velocity/end-diastolic velocity ratio of the umbilical artery (UA), PI of the left uterine artery, and PI of the middle cerebral artery were all <5th centile; the systolic/atrial wave ratio of the ductus venosus was 50th to 95th centile. Then the examination focused on the umbilical cord, excessive torsion of the umbilical cord and 2 hyperechoic lesions in 1 UA were observed. Only 1 UA was detected in the bladder section by color Doppler flow, whereas previous ultrasound screening results in the mid-trimester showed 2 umbilical arteries in the same section. Additionally, electronic heart rate monitoring revealed a noncontractile stimulation test abnormality.

**Diagnoses:**

UAT with fetal and hemodynamic changes were observed.

**Interventions:**

Urgent cesarean section was performed 2 days later after comprehensive prenatal counseling.

**Outcomes:**

The infant was born alive at 1740 g, and Apgar scores were 9-10-10 in 1-5-10 minutes respectively. Postoperative recovery of the pregnant woman was unremarkable.

**Lessons:**

Targeted Doppler evaluation and timely delivery are helpful for the diagnosis of UAT and obtaining a fine pregnancy result.

## 1. Introduction

Umbilical artery thrombosis (UAT) is a rare abnormality of the umbilical cord (UC) that may be life-threatening to the fetus.^[1–3]^ Its occurrence is difficult to predict due to the lack of comprehensive reporting of precise risk factors. It is difficult to monitor the disease because UC abnormalities are often missed owing to the fetal position and experience of the ultrasound doctor, and it is easily misdiagnosed as a single UA. Choosing an optimal delivery time to achieve good perinatal outcomes is an additional challenge following diagnosis. La Verde et al reported that fetal Doppler and uterine artery blood flow volume could be helpful in high-risk pregnancy management.^[4]^

Here, we present a case of prenatally suspected and ultrasound diagnosed UAT due to hemodynamic changes. Timely delivery was performed based on targeted Doppler evaluation and the high resolution flow (HR-Flow) evaluation of the placenta to obtain a fine pregnancy result. We also discuss and summarize this case based on a literature review.

## 2. Case presentation

A 35-year-old gravida 6, para 1 pregnant woman was admitted to our hospital in December 2024 with reduced fetal movement at 32 weeks of gestation. Physical examination revealed a uterine height of 32 cm, an abdominal circumference of 98 cm, and a body mass index of 29.9 of the women. The vital signs of the woman and fetal heart rate were within the normal ranges. This pregnancy requires L-thyroxine owing to hypothyroidism. Noninvasive prenatal testing suggested a low-risk pregnancy. The mid-trimester fetal ultrasound scan was normal. A male infant weighing 3600 g was delivered via cesarean section 16 years ago because of fetal distress.

**Figure 1.**
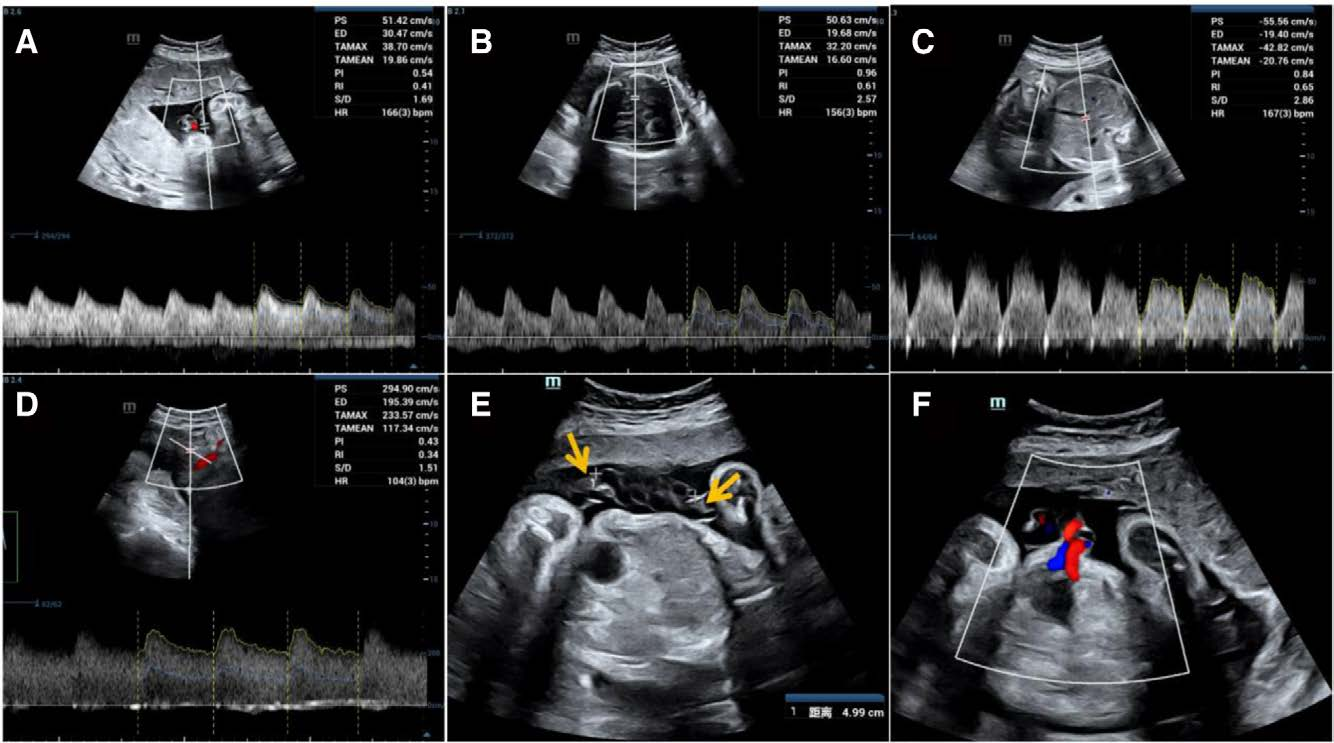
Hemodynamics arrangement of the UAT fetal. The values of UA-PI, RI, and S/D (A), MCA-PI (B), left UtA PI (C) were all <5th centile for gestational age of 32 weeks. The value of systolic/atrial wave ratio of ductus venosus (D) was in the 50th to 95th centile for gestational age of 32 weeks. (E) The excessive torsion of UC (the umbilical coiling index was 3/4.99 = 0.6) and 2 hypoechoic thrombosis (orange arrows) were observed through 2-dimensional ultrasound. (F) Only 1 UA showed in the section of bladder through color Doppler ultrasound. MCA = middle cerebral artery, PI = pulsatility index, RI = resistance index, S/D = systolic/diastolic, UA = umbilical artery, UAT = umbilical artery thrombosis, UC = umbilical cord, UtA = uterine artery.

Prenatal ultrasound estimates the fetal weight of approximately 1690 g based on biparietal diameter of 7.7 cm, head circumference of 27.9 cm, abdominal circumference of 27.6 cm, and femur length 5.8 cm, which was <10th centile for gestational age of 32 weeks. The amniotic fluid index was normal. Doppler ultrasound showed the following parameters of the umbilical artery (UA): pulsatility index (PI) = 0.54, resistance index (RI) = 0.41, and peak systolic velocity/end-diastolic velocity (S/D) ratio = 1.69, all parameters were <5th centile for a gestational age of 32 weeks. The parameters of the uterine artery (UtA) were as follows: left UtA PI = 0.43, RI = 0.34, S/D = 1.5, right UtA PI = 0.54, RI = 0.41, S/D = 1.7. The middle cerebral artery (MCA) had PI = 0.96, RI = 0.61, and S/D = 2.57. The left-UtA PI and MCA-PI were <5th centile at a gestational age of 32 weeks. The systolic/atrial wave ratio of the ductus venosus was 2.86, which was in the 50th to 95th centile (Fig. 1A–D).

**Figure 2.**
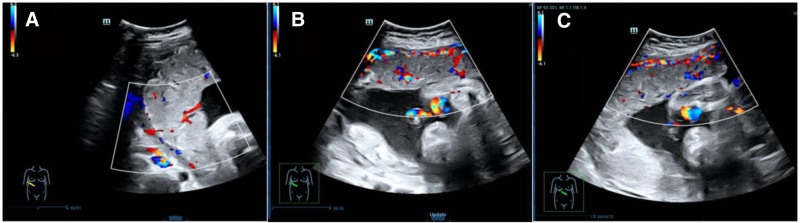
The vascularization of placenta. (A) The villous tree-like structure in the middle region of placenta. (B and C) The sparse strip-shaped blood flow in the left and right regions of placenta.

The examination was then focused on the UC, on 2-dimensional ultrasound, there were 3 vessels in the UC, the umbilical coiling index was 0.6, and 2 hypoechoic lesions were observed in one of the umbilical arteries. There was only 1 UA in the bladder section showed by color Doppler flow (Fig. 1E–F). Additionally, we observed villous tree-like structures only in the middle region of the placenta using HR-Flow imaging with a 2 mm sampling volume, 5 to 7 cm/s speed, and 0° to 60° angle,^[5]^ and sparse strip-shaped blood flow was observed in the left, right, upper, and lower regions of the placenta (Fig. 2).

In the clinic, electronic heart rate monitoring revealed a noncontractile stimulation test abnormality. The plasma D-dimer was 993 ng/mL and fibrinogen was 6.07 g/L, which were higher than the normal range. According to the strong maternal request, expectant treatment was performed under close monitoring, while corticosteroids were used to promote fetal lung maturity.

**Figure 3.**
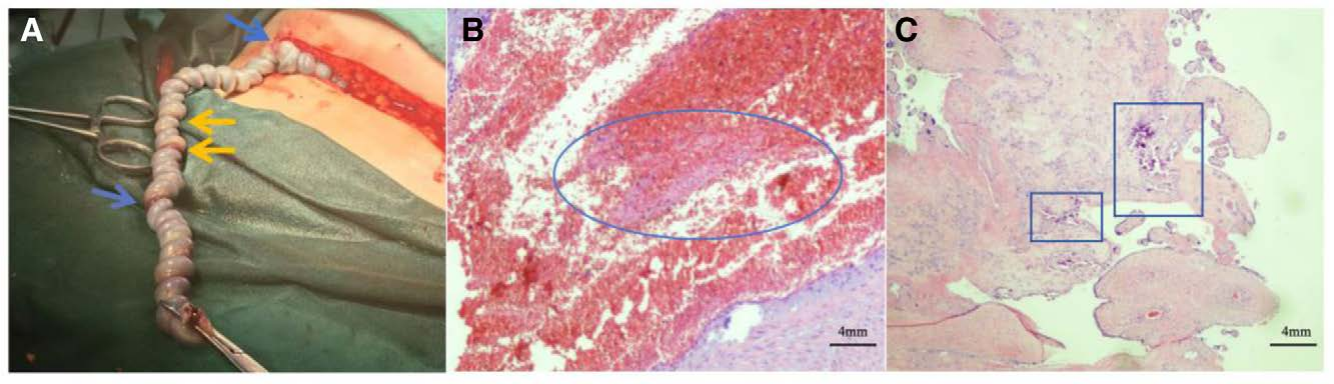
Postnatal examination of UC and histology of placenta. (A) The excessive torsion of UC and the thrombosis presented by gross observation (both the orange and blue arrows, and the orange arrows correspond to arrows in Figure 1E, which were observed by prenatal ultrasound). (B) The thrombosis confirmed by pathology (circle). (C) The focal areas of calcification within the placenta (square). Hematoxylin and eosin, ×20 magnification. UA = umbilical artery, UC = umbilical cord.

The next day, the fetal biophysical score was 4, and a detailed late pregnancy ultrasound examination was conducted to exclude associated anomalies according to the ISUOG practice guidelines.^[6]^ The third day, fetal heart monitoring showed prolonged deceleration, plasma D-dimer was 22,383 ng/mL (Table 1), the patient underwent emergency cesarean section, and delivered a girl weighing 1740 g with an Apgar score of 9-10-10. Postnatal examination confirmed thin and excessive torsion of UC with congestion in multiple areas. The diagnosis of UAT was histologically confirmed, and under the microscope, the placental villi were consistent with changes in the mid to late stages of pregnancy, accompanied by focal calcification and increased deposition of cellulose between the placental villi and beneath the chorionic plate. Cellulose deposition and focal inflammatory cell infiltration were observed in fetal membranes (Fig. 3). Both the mother and baby were discharged in good health on postpartum day 5.

## 3. Discussion

The incidence of UAT is 0.0025% to 0.8%,^[7–9]^ and is generally associated with poor perinatal outcomes, such as severe intrauterine growth restriction, fetal intrauterine distress, and even stillbirth. It had been reported that approximately 10% of stillbirths are affected by UAT.^[10,11]^ The pathogenesis of UAT are remains unclear, and many risk factors, such as the UC torsion, abnormal UC insertion, maternal coagulation abnormalities and complications, and vascular endothelial inflammation, have been reported to be related to UAT.^[12]^ In the case series of Wu et al,^[1]^ the gestational diabetes mellitus and UC abnormalities were the independent risk factors for the development of UAT. However, comprehensive studies on this topic are lacking. In our case, UC torsion and blood hypercoagulability were observed, which may have been the cause of the UAT.

### 3.1. Diagnosis of the UAT

Although ultrasound is the preferred method for detecting UC abnormalities, prenatal diagnosis of UAT is challenging because of its low incidence and the fact that many pregnant women have no obvious symptoms or signs. Many cases have been confirmed by pathological examination of postpartum UC. In some cases, the typical ultrasound finding is a single umbilical artery (SUA), which can be identified by Doppler ultrasound in the section of the bladder. However, UAT should be differentiated from SUA because it requires the exclusion of additional structural and/or chromosomal abnormalities,^[13]^ and UAT needs to focus on the evaluation of fetal intrauterine status. Therefore, when considering SUA for the first time, especially during mid to late pregnancy, it is necessary to confirm whether the previous ultrasonic examinations showed bilateral umbilical arteries.

This case was first found hemodynamic change of UA, then through the targeted Doppler evaluation, and comprehensive and standardized Doppler evaluation which reported by Tu et al,^[10]^ the UAT was founded. Additionally, histological placental data were collected, which maybe the merit of this case.

### 3.2. Hemodynamic changes of UAT

The hemodynamic characteristics of fetuses and placentas with UAT are not fully understood. Changes in fetal hemodynamics observed by Doppler ultrasound may be an early warning signal.^[10,14,15]^ This case focused on the UC after identifying abnormal blood flow waveforms in the UA. The thrombus in one of the UA can result in compensatory hemodynamically of the remaining artery, and the distal resistance is then reduced. In addition, UAT can result in reduced oxygen supply, leading to fetal self-protection through the redistribution of cerebral blood flow, increasing diastolic flow, and reducing MCA-PI.

In our case, we also observed the hemodynamic features of the placenta with HR-Flow, only in the middle region of the placenta can see the villous arborescence blood flow, He et al reported that it means a decrease in placental blood perfusion,^[5]^ which may be associated with the hypoxic condition caused by UAT leading to endometrial swelling and endothelial necrosis, ultimately resulting in the occlusion of placental villi vessels,^[16]^ and further leads to an increase in perfusion and a decrease in the PI of the UtA. This indicates that the blood flow change in the UA, MCA, and placenta may not be gradual process of change.

### 3.3. The management of the pregnancy with UAT

There are no standard guidelines for the management of UAT. Pregnancy management is challenging when UAT is diagnosed in the second trimester or at an early term. In the late trimester, most patients choose cesarean section to terminate the pregnancy shortly after diagnosis. In recent years, some pregnant women and doctors have chosen protective and expectant management patterns^[1,17,18]^ especially for fetuses <32 weeks old, and have found that compared to the emergency treatment group, the expected fetal outcomes were not worse.

However, UAT can cause sudden intrauterine death of the fetus, which remains unpredictable, and close monitoring, such as fetal movement, ultrasound, coagulation indicators, and electronic fetal heart monitoring, is required when choosing expectant management while preparing for an emergency cesarean section. To our knowledge, controversy regarding how often the examination should be conducted before terminating pregnancy still exists. In a case series by Tu et al, DV-PI for veins was an independent risk factor for adverse pregnancy outcomes.^[10]^

In this case, according the hemodynamic parameters of the DV and the presence of villous tree-like structure in the middle region of the placenta, which we believe the baby still has a certain tolerance and the placenta still has a certain compensatory ability, and we followed up the pregnant women with intensive fetal heart monitoring and daily ultrasound due to abnormalities in the MCA and UA. Despite the mother’s strong request, it only prolonged the gestational age by 2 days due to fetal intrauterine distress indicated by biophysical scores and prolonged deceleration in fetal heart rate monitoring.

One limitation of this study was that it could not determine the exact time of UAT occurrence or understand its complete hemodynamic changes. Another limitation was the absence of prior serial Doppler before admission and no known complete history of a coagulation profile.

**Table 1:** The visual timeline for the important diagnostic and management steps.

	32W^+0^	32W^+1^	32W^+2^
Pregnant woman	Feel reduced fetal movement Be hospitalized Required expectant treatment	Refused cesarean section	Agreed and underwent emergency cesarean section
Clinician	Suggest cesarean section Promote fetal lung maturity	Suggest cesarean section	Emergency cesarean section
Electronic heart rate monitoring	Noncontractile stimulation test abnormality	Noncontractile stimulation test abnormality	Prolonged deceleration
Lab	Plasma D-dimer: 993 ng/mL Fibrinogen: 6.07 g/L		Plasma D-dimer: 22,383 ng/mL
Ultrasound	UA: RI, PI and S/D all <5th centile* UtA: left-UtA PI <5th centile* MCA: PI < 5th centile* DV: Systolic/atrial wave ratio in the 50th to 95th centile* UC: (1) 3 vessels, while only 1 UA in the bladder section shown by color Doppler flow (2)Umbilical coiling index was 0.6 (3)2 hypoechoic lesions were observed in one of the umbilical arteries Placenta: HR-Flow shows the villous tree-like structures only in the middle region of placenta	The fetal biophysical score: 4 score	

DV = ductus venosus, HR-Flow = high resolution flow, MCA = middle cerebral artery, PI = pulsatility index, RI = resistance index, S/D = systolic/diastolic, UA = umbilical artery, UC = umbilical cord, UtA = uterine artery.

* Compared with the reference range of 32 wk.

## 4. Conclusion

Although rare, UAT should be considered if there is evidence or relevant high-risk factors. Early identification of the UAT and comprehensive color Doppler evaluation provide a basis for perinatal health care in women.

## Author contributions

**Conceptualization:** Gang Li.

**Data curation:** Yeping He.

**Funding acquisition:** Jianfeng Guo.

**Investigation:** Xiufeng Yin.

**Methodology:** Gang Li.

**Project administration:** Jianfeng Guo.

**Supervision:** Yeping He, Jianfeng Guo.

**Validation:** Yeping He.

**Visualization:** Gang Li.

**Writing – original draft:** Gang Li, Xiufeng Yin, Yeping He.

**Writing – review & editing:** Yeping He.
